# Infective Endocarditis Following Post-COVID Organizing Pneumonia Complicated by Multiple Splenic Abscesses and Glomerular Nephritis

**DOI:** 10.7759/cureus.45860

**Published:** 2023-09-24

**Authors:** Mohamed Jazeer, Mayurathan Pakkiyaretnam

**Affiliations:** 1 Internal Medicine, Teaching Hospital Batticaloa, Batticaloa, LKA; 2 Internal Medicine, Srijayawardanapura University, Batticaloa, LKA; 3 University Medical Unit, Teaching Hospital Batticaloa, Batticaloa, LKA; 4 Clinical Sciences, Faculty of Health-Care Sciences, Eastern University of Sri Lanka, Batticaloa, LKA

**Keywords:** glomerular nephritis, vasculitis, pneumonia, infective endocarditis, covid-19

## Abstract

The association of glomerular nephritis and infective endocarditis with liver abscesses is a clinically complex entity that often makes the diagnosis challenging. Here, we report a case of a 50-year-old woman who presented with a febrile illness of two weeks' duration along with myalgia and malaise of four days' duration. She had a background history of well-controlled type 2 diabetes mellitus for five years with a past history of ischemic heart disease diagnosed five years ago. At the time of presentation, she was on long-term steroids for post-coronavirus disease (COVID) organizing pneumonia diagnosed three months back. With serial investigations, she was found to have subacute bacterial endocarditis with multiple liver and splenic abscesses. She was managed with antibiotics as per local protocols after which she made a successful recovery of her clinical status. The uniqueness of this case is the development of rare complications of subacute bacterial endocarditis in the background of immunosuppression.

## Introduction

Infective endocarditis (IE) is defined as an infection of the endocardial surfaces of the heart, primarily of one or more heart valves, the mural endocardium, or a septal defect. Its intra-cardiac effects include severe valvular insufficiency, intractable congestive heart failure, and myocardial abscesses. If left untreated, IE is inevitably fatal [1].

The use of the standardized Duke classification, which combines two major criteria (microbiology and imaging) and five minor criteria, aids in the diagnosis of infective endocarditis (fever, predisposing cardiomyopathy, vascular phenomena, immunological phenomena, microbiological evidence [2].

Classification of IE is important for disease management. There are numerous classifications for IE, out of which classifying IE based on the causative agent plays a major role. The pathogens that are responsible for causing endocarditis include *Enterococcus spp, Streptococcus bovis, Staphylococcus aureus, *etc.The other factors that are responsible for causing infective endocarditis include age, gender, the type of valve, and the characteristics of the injured valve.

*Enterococcus spp* and *Streptococcus bovis* are commonly found in the elderly population who are affected by IE. *Streptococcus bovis* has a higher predilection toward the mitral valve and is found to be the major pathogen causing mitral valve endocarditis. Considering the type of valve, *Streptococcus spp.* commonly affect the native valves while coagulase-negative staphylococci and *Coxiella burnetii* are causative pathogens for intracardiac prosthetic valve endocarditis.* S. bovis *and *Staphylococcus aureus* are prominent species associated with previously healthy valves while oral streptococci cause IE mainly in patients with previous valvular damage [3].

*Enterococcus faecalis* is the third most frequent causative organism with more healthcare-associated infections and increasing incidence of antimicrobial resistance with high morbidity and mortality [4]. The typical clinical presentation is a subacute course with fever, malaise, and generalized aches, difficult to distinguish from other, more common diseases [5]. *Enterococcus* *faecalis *causing infective endocarditis following coronavirus disease 2019 (COVID-19) infection has not been reported yet.

## Case presentation

A 50-year-old woman presented with a two-week history of fever unresponsive to antipyretics, associated with myalgia and malaise of four days duration. She had a past medical history of ischemic heart disease and type 2 diabetes mellitus diagnosed five years back for which she had been on regular follow-ups. At the time of presentation, she had no macro or microvascular complications of diabetes mellitus and was on oral hypoglycemic therapy. Despite the history of ischemic heart disease, she was doing well until three months prior to this presentation when she was diagnosed with COVID pneumonia and was diagnosed having active fibrotic changes, post-COVID organizing pneumonia with more than 70% lung involvement. Since then she has been suffering from difficulty in breathing for the past three months, restricting her activities of daily living. Since the diagnosis of COVID pneumonia three months back, she was on a tapering regime of oral steroids when she presented with the above symptoms.

**Figure 1:**
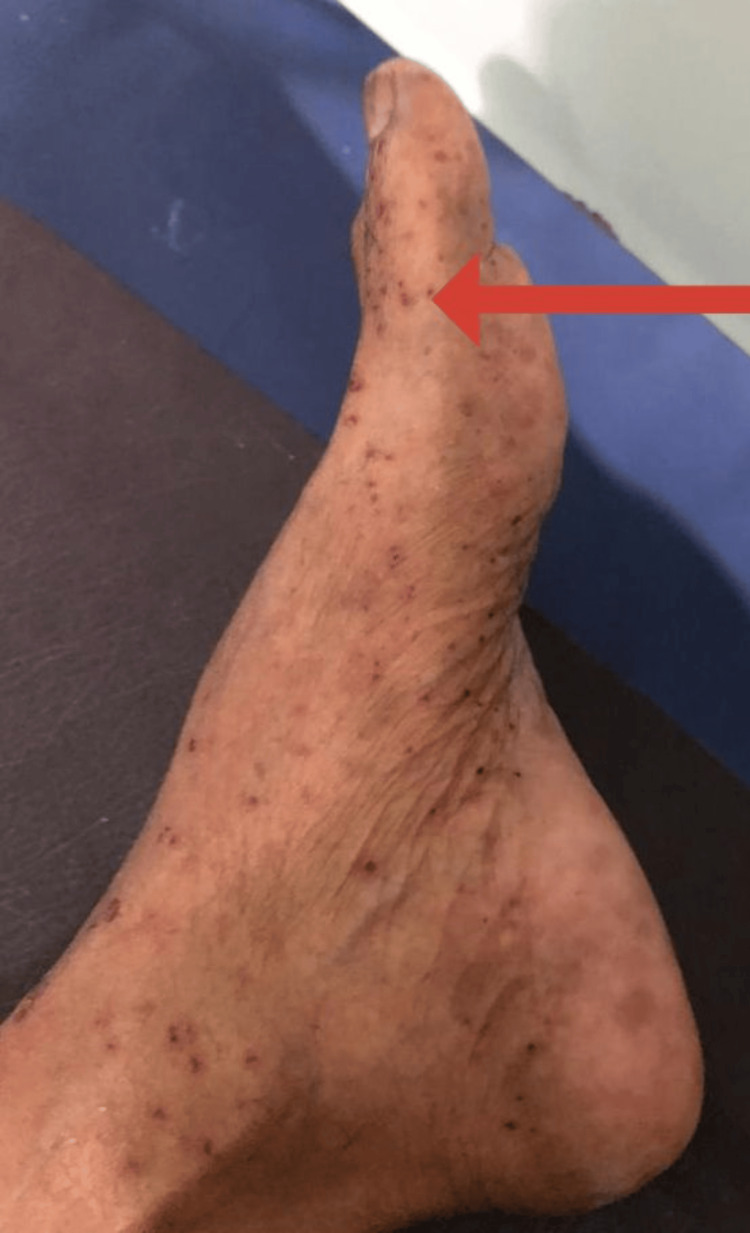
Vasculitic rash secondary to septic emboli

**Figure 2:**
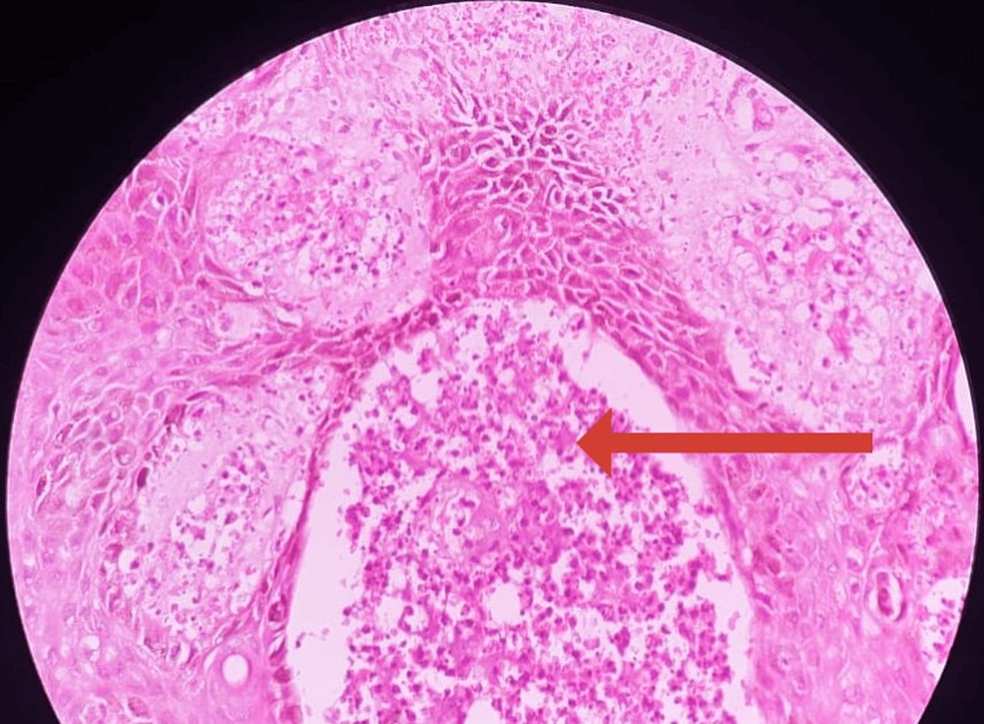
Microscopy showing secondary vasculitis due to septic emboli

On examination, she was febrile, tachypnoeic, and tachycardic. She had a vasculitic rash with multiple petechiae and palpable purpura confined to symmetrical bilateral lower limbs (Figure 1). Splinter hemorrhages were noted on bilateral toenails. No subcutaneous nodules, digital gangrene, or ulcers were noted. Her erythrocyte sedimentation rate (ESR) and C-reactive protein (CRP) were significantly high. Two blood cultures that were obtained 12 hours apart were positive for enterococci. An ultrasound scan of the abdomen was done in view of arriving at a diagnosis, and it showed multiple splenic abscesses. A 2-dimensional echocardiogram revealed an ejection fraction of 45-50%, rupture of chorda tendinae associated with the mitral valves, and a small pericardial effusion along with multiple vegetations in association with anterior mitral valve leaflets. The vasculitic rash was evaluated by the dermatologist and histopathologist through punch biopsy specimens taken from bilateral lower limbs (Figure 2). The investigations that were done are shown in Table 1.

**Table 1:** Investigations

Investigations		Day 01	Day 05	Day 10	On Discharge	Reference Range
White cell count (*10^9^/L)		8.24	20.5	12.38	9.12	4 – 11
Neutrophils (%)		75%	90%	84%	88%	50 -70
Lymphocytes (%)		11.60%	7%	11.20%	6.90%	20 - 40
Eosinophils (%)		6.70%	0.30%	1.60%	3.30%	1 – 5
Hct (%)		3.05	3	4.2	3.71	36 - 48
Hemoglobin (g/dL)		8	7.7	9.1	10.1	12 – 14
Platelets (*10^9^/l)		158	301	402	163	150- 400
Erythrocyte sedimentation rate (ESR) (mm in 1^st^ hr)		12	-	-	-	< 20
Serum sodium (mmol/L)		138	135	139	143	135 – 145
Serum potassium (mmol/L)		3.5	3	3.3	3.5	3.5 – 5.5
Aspartate transaminase (AST) (U/L)		30	39	21	40	< 40
Alanine transaminase (ALT) (U/L)		31	37	29	24	< 40
Alkaline phosphatase (ALP) (IU/L)		272	242	197	164	44 - 147
Total protein (g/L)		60	53	58	69	60 - 83
Serum albumin (g/L)		22	18	25	32	34 -54
Serum globulin (g/L)		38	37	40	37	20 - 35
Serum creatinine (µmol/L)		55	59	105	145	53 - 97
C-reactive protein (CRP) (mg/dL)		177	294	106	1.7	< 5
Total bilirubin (mg/dl)		8.6	22.9	11.6	4.8	0.1 – 1.2
Direct bilirubin (mg/dl)		13.7	-	-	4.5	0.1 – 0.3
Indirect bilirubin (mg/dl)		9.2	-	-	7.1	0.2 - 0.8
Procalcitonin (µg/l)		0.084	-	-	-	< 0.05
Urine protein creatinine ratio (mg/g)		296	1531	-	222	150 - 500
International normalized ratio (INR)		1.19	-	-	0.88	<1
Ultrafiltration rate (UFR)	Albumin	NIL	++	TRACE	+	
	Red cells	20	-	-	-	
	Pus cells	field full	moderately field full	field full	moderately field full	

She was confirmed negative for antineutrophilic cytoplasmic antibody (ANCA)-associated vasculitis. Her urine analysis was positive for proteinuria and gross hematuria, however, her renal functions were normal. Based on the clinical history, vegetations detected on transthoracic echocardiography with positive blood cultures for enterococci, the diagnosis of subacute infective endocarditis (SABE) was beyond reasonable doubt, hence the renal manifestations of glomerulonephritis and vasculitis were attributed to membranoproliferative type glomerulonephritis, which can occur as a consequence of SABE. Therefore, a renal biopsy was not carried out, as the clinical diagnosis was apparent. Her computerized tomography pulmonary angiogram findings suggested no major pulmonary embolism, however, commenting on segmental arteries regarding emboli was challenging. There was also a ground-glass appearance in the lower segment of the lung field suggestive of post-COVID organizing pneumonia. Her procalcitonin level was high, suggestive of ongoing inflammation.

With the support of the aforementioned features, positive blood cultures, and 2D echo findings, the diagnosis of subacute bacterial infective endocarditis was made and the patient was managed according to the national antibiotic guidelines. He was initiated on treatment with intravenous (IV) crystalline penicillin 6 MU 6 hourly, and IV gentamycin 50 mg 8 hourly. IV Albumin was added further to her drug regime along with IV frusemide. Prednisolone was continued as per the preplanned tapering regimen. Her routine medications were continued as part of her therapy regimen. The patient made a complete recovery after the completion of IV Antibiotics for 28 days. A 2-dimensional echocardiogram after the completion of the treatment showed healed vegetation. Upon discharge, respiratory follow-up was arranged for the post-COVID organizing pneumonia.

## Discussion

Enterococcus endocarditis is frequently reported in older men in a subacute fashion [6,7]. Our female patient was on a tapering regime of oral steroids for post-COVID organizing pneumonia prior to the index admission. The background history of diabetes mellitus causes immunosuppression and the administration of steroids likely predisposed the patient to infective endocarditis.

Acute renal failure and splenic abscesses are less common in IE [8]. However, our patient developed both of these complications, which could be due to the relatively severe immunosuppression with steroids following COVID infection.

Successful treatment requires appropriate antibiotic therapy. Empirical therapy should be initiated as early as possible. The choice of definitive antibiotic therapy is based on the causative microorganism and its antibiotic susceptibility [9]. Surgery may need to be considered in selected patients with valve dehiscence, perforation, rupture, or fistula, or a large perivalvular abscess, vegetation more than 10 mm, and prosthetic valve endocarditis [10].

## Conclusions

Early diagnosis and treatment of infective endocarditis are paramount, especially in patients with pre-existing cardiac disease. As evident from this case, the patient had a past history of ischaemic heart disease, a background history of type 2 diabetes mellitus, and a history of post-COVID organizing pneumonia for which she was on steroids. All these conditions led to an immunosuppressive state that led to the development of less common complications like acute renal failure and splenic abscesses along with sub-acute bacterial endocarditis in this patient. With the given clinical context, the diagnosis of glomerulonephritis is apparent, hence the need for renal biopsy to determine the exact histological type is not mandatory for patient management and the management should be done as per local antibiotic protocols. Of noteworthy value in this case is the importance of active surveillance for the development of possible complications in patients who are on immunosuppressant medication such as corticosteroids for another condition.
